# Scleraxis expressing scleral cells respond to inflammatory stimulation

**DOI:** 10.1007/s00418-021-01985-y

**Published:** 2021-05-08

**Authors:** Ghada Atta, Falk Schroedl, Alexandra Kaser-Eichberger, Gabriel Spitzer, Andreas Traweger, Ludwig M. Heindl, Herbert Tempfer

**Affiliations:** 1 Department of Ophthalmology, Faculty of Medicine, University of Cologne, University Hospital Cologne, Cologne, Germany; 2 Institute of Tendon and Bone Regeneration, Spinal Cord Injury and Tissue Regeneration Center Salzburg, Paracelsus Medical University, Salzburg, Austria; 3 Austrian Cluster for Tissue Regeneration, Vienna, Austria; 4 Center for Anatomy and Cell Biology, Institute of Anatomy and Cell Biology - Salzburg, Paracelsus Medical University, Salzburg, Austria; 5 Center for Integrated Oncology (CIO) Aachen-Bonn-Cologne-Düsseldorf, Cologne, Germany

**Keywords:** Sclera, Tendon, Scleraxis, Scleritis model

## Abstract

The sclera is an ocular tissue rich of collagenous extracellular matrix, which is built up and maintained by relatively few, still poorly characterized fibroblast-like cells. The aims of this study are to add to the characterization of scleral fibroblasts and to examine the reaction of these fibroblasts to inflammatory stimulation in an ex vivo organotypic model. Scleras of scleraxis-GFP (SCX-GFP) mice were analyzed using immunohistochemistry and qRT-PCR for the expression of the tendon cell associated marker genes *scleraxis* (SCX), *mohawk* and *tenomodulin*. In organotypic tissue culture, explanted scleras of adult scleraxis GFP reporter mice were exposed to 10 ng/ml recombinant interleukin 1-ß (IL1-ß) and IL1-ß in combination with dexamethasone. The tissue was then analyzed by immunofluorescence staining of the inflammation- and fibrosis-associated proteins IL6, COX-2, iNOS, connective tissue growth factor, MMP2, MMP3, and MMP13 as well as for collagen fibre degradation using a Collagen Hybridizing Peptide (CHP) binding assay. The mouse sclera displayed a strong expression of scleraxis promoter-driven GFP, indicating a tendon cell-like phenotype, as well as expression of *scleraxis*, *tenomodulin* and *mohawk* mRNA. Upon IL1-ß stimulation, SCX-GFP+ cells significantly upregulated the expression of all proteins analysed. Moreover, IL1-ß stimulation resulted in significant collagen degradation. Adding the corticosteroid dexamethasone significantly reduced the response to IL1-ß stimulation. Collagen degradation was significantly enhanced in the IL1-ß group. Dexamethasone demonstrated a significant rescue effect. This work provides insights into the characteristics of scleral cells and establishes an ex vivo model of scleral inflammation.

**Supplementary Information:**

The online version contains supplementary material available at 10.1007/s00418-021-01985-y.

## Introduction

The sclera forms around 85% of the rigid outer tunic coat of the human eyeball, which consists of three anatomical layers: (1) the superficial vascularized episclera which contains a tight network of blood vessels, (2) the scleral stroma resembling the main part, which is mainly avascular, and (3) the lamina fusca, a thin pigmented layer which is located above the uvea, an also strongly pigmented layer below the sclera. The sclera is a remarkably resistant, stable connective tissue that performs various functions critical to vision: its primary role includes providing a firm and stable substrate for the retina to protect retinal and other internal structures of the eye from damage due to their mechanical vulnerability (Boote et al. 2020). Physiologically, the adult sclera shows only a superficial network of blood vessels but lacks lymphatic vessels, thereby creating a lymphatic-free border to the inner eye (Schlereth et al. 2019), ultimately contributing to the ocular immune privilege. This vascular privilege is actively regulated by balancing anti- and proangiogenic factors expressed by cells within the sclera (Atta et al. 2020). Despite sclera being considered a quiescent tissue in healthy state, pathologic conditions such as injuries or tumours trigger tissue responses leading to matrix disruption and cellular activation (Harper and Summers 2015). Consequently, the scleral vascular privilege is compromised and secondary ingrowth of intraocular lymphatic vessels with a significant number of associated LYVE-1 + macrophages takes place, invading the sclera and the inner eye (Schlereth et al. 2014a, b). This mechanism supports wound healing, defense against invading microorganisms and autoimmune reactions against intraocular antigens (Schlereth et al. 2014a, b).

Regarding the scleral cellular phenotype(s), there is still a lack of characterization of scleral resident cells. Except for the innermost layer of the sclera connected to the choroid, the lamina fusca, most regions of the sclera are sparsely populated by cells. The vast majority of resident cells of the scleral stroma are defined as “fibrocytes”, transforming to an active fibroblast upon insult. While fibroblasts are responsible for synthesis of all scleral ECM components, such as collagen, proteoglycans, and elastic fibres, they also respond to mechanical stimuli from their surrounding ECM, potentially inducing matrix remodelling and undergoing proliferation. Under mechanical stimulation, the expression of thrombospondin-1, HINT1, vimentin, actinin, and α-smooth muscle actin was shown to be increased in scleral fibroblasts (Boote et al. 2020; Oglesby et al. 2016). In an RNAseq approach, cultured scleral fibroblast cells derived from healthy human eyes were demonstrated to express fibronectin, collagen I, III, and VI and further also MMP2 under non- pathologic culture conditions (Löbler et al. 2013).

Under inflammatory conditions, the scleral matrix is severely affected, resulting in structural changes and tissue disintegration with necrosis of the outer tunic, as it is the case in scleritis (Watson and Romano 2014). While this disease is associated with severe pain and possibly permanent loss of vision, the mechanisms of this disease remain unclear (Hankins and Margo 2019). In most of the cases, autoimmune mechanisms are likely the underlying cause, while post-traumatic and iatrogenic cases have also been reported. However, generally other aetiologies remain enigmatic and the underlying mechanisms are poorly understood. The few existing animal models are not quite satisfying (Wakefield et al. 2013; Hankins and Margo 2019), as they partly use non-physiologic types of scleritis induction such as ovalbumin application. Most of the human data derive from histo-pathological investigations.

Interestingly, tendon tissue shares various similarities with the sclera; they are mainly composed of collagen-rich, extracellular matrix, built up and maintained by relatively few, spindle shaped fibroblasts. They are sparsely vascularized in a healthy state, whereas a hallmark of tendon disease includes hypervascularity (Tempfer and Traweger 2015). Similar to scleritis, degenerative tendon pathologies are often associated with systemic inflammatory diseases such as psoriatic arthritis (Kaeley et al. 2018). Similar to the sclera, tendon cells are still rather poorly characterized, and progress is in part hampered by the lack of truly specific marker proteins. Generally, tenomodulin (Tnmd), thrombospondin 4 (TSP-4), tenascin C (TNC) and collagens type 1 and 3 are commonly used for identification and characterization of tendon fibroblasts. Tenascin C, tenomodulin and thrombospondin 4 were also found to be expressed in various tissues, the latter two also in the sclera (Oshima et al. 2003; Si et al. 2003; Dex et al. 2016). The most accepted marker for tendon cells, however, is the basic helix loop helix transcription factor scleraxis. The loss of the scleraxis gene leads to disrupted tendon development with matrix disorganization and motion deficits (Murchison et al. 2007). Despite not being a master regulator of tendon development, fate mapping and scleraxis-GFP reporter mouse models underline the importance of scleraxis as a marker for tendon cells (Pryce et al. 2007). In the eye, expression of scleraxis has been shown in the tendons attaching to the ocular muscles (Grenier et al. 2009); however, examination of its expression in the sclera is still lacking.

As tendon and sclera share a large variety of properties, such as collagen rich matrix, low cell density, sparse vascularization, and high mechanical stress under physiological conditions, it is the aim of the present study to further characterize scleral cells regarding their expression of tendon associated markers in adult and embryonic mouse eyes and to examine the response of these cells to an inflammatory environment in an organotypic approach.

## Materials and methods

### Animals

All procedures involving animals were carried out in an approved animal facility by authorized staff and were in accordance with all relevant Austrian laws. As only tissue from euthanized animals was used, no further ethics approval was required. Scleraxis-GFP (ScxGFP) reporter mice (Pryce et al. 2007) were kindly shared by Prof. Denitsa Docheva (University of Regensburg, Germany). All animals were acclimatized to standard laboratory conditions (12-h light, 12-h dark cycle) and given free access to rodent chow and water. Tissue was harvested from 12 week old mice. For generation of E17 mouse embryos, embryonic day 0.5 (E0.5) was specified as the day when the experimenter confirmed the presence of a vaginal plug. Embryos were obtained from the pregnant mice, which were sacrificed by cervical dislocation. For qRT-PCR analyses, 12 week old male C57Bl/6 mice were obtained from Janvier Labs (Le Genest-Saint-Isle, France).

### *Organotypic sclera* inflammation model

For the experiment, three female Tg (Scx-GFP) 1 Stzr mice (3 months of age) were sacrificed by cervical dislocation. Subsequently, the eyes were dissected, and all non-scleral tissue was removed under sterile conditions. Sclera has been divided into three segments with the optic nerve in the centre and three cuts reaching from the centre to 12, 4 and 8 o’clock direction. Obtained segments/triangles were further subdivided in tangential cuts from centre to midline periphery and sagittal cross sections (i.e., at 2, 6 and 10 o’clock) obtained and immediately transferred to 6 well plates containing 10% fetal bovine serum in minimum essential medium. One group was further supplemented for 3 days with 10 ng/ml IL1-ß (PeproTech, Vienna, Austria), or 10 ng/ml IL1-ß and 100 nM dexamethasone (Sigma-Aldrich, Vienna, Austria), respectively. These concentrations were chosen according to several published protocols on tendon cells (e.g. Gehwolf et al. 2019; Mousavizadeh et al. 2015). The tissue pieces were incubated for 3 days at 37 °C and at 5% CO_2_, with daily media change. The tissues were then fixed in 4% paraformaldehyde overnight, washed three times in PBS and processed for further cryosectioning (Supplementary Fig. 1).

### Preparation of tissue sections

Mouse eyes were fixed in phosphate-buffered saline (PBS) containing 4% paraformaldehyde for 12 h at 4 °C. Following several rinses in PBS and cryo-preservation in PBS containing 30% sucrose, tissues were embedded in cryomedium (Surgipath Cryogel^®^, Leica Microsystems, Vienna, Austria) and 15 μm cryosections were prepared (CM1950, Leica, Vienna, Austria).

### Detection of denatured collagen

To detect damaged or denatured collagen, collagen hybridizing peptide (CHP, 3helix Inc, Salt Lake City, Utah, USA) was applied onto cryo-sections of organotypic cultured mouse sclera, according to the manufacturers’ protocol. This peptide specifically binds to unfolded collagen chains, thus detecting degraded collagen only.

Briefly, the sections were incubated with 5 µM CY3-labelled CHP and 1 µg/ml 4′,6-diamidino-2-phenylindol dihydrochloride (DAPI) at 4 °C in a humified chamber for 18 h. Confocal images were acquired on a confocal laser scanning microscope (LSM710, Zeiss, Vienna, Austria) using a 20× objective.

For semi-quantitative analysis of expression intensity, the images were analysed by ImageJ software, calculating % area with CHP positive signal (Fig. 2).

### qRT-PCR analysis

Total RNA was isolated from sclera, hind limb digital flexor tendons and optic nerve from 12 months old male C57Bl/6 mice (*n* = 4 animals, tissues pooled from left and right side) using TRI^®^ Reagent (Sigma-Aldrich) according to the manufacturer’s protocol. RNA yield was quantified by Nanodrop 2000C (Thermo Fisher Scientific, Vienna, Austria), RNA integrity was verified using an Experion Automated Electrophoresis system (Bio-Rad, Munich, Germany). A minimum requirement of RNA quality indicator (RQI) > 7.5 was chosen.

qRT-PCR was performed as described by Gehwolf et al. (2019) using TaqMan® assays from Integrated DNA Technologies (Coralville, IA, USA). Amplification conditions were 50 °C for 2 min, 95 °C for 10 min, followed by 40 cycles of 95 °C for 15 s and 60 °C for 1 min. All samples were run in duplicate. CQ values were analyzed using qBasePlus v. 2.4 (Biogazelle NV, Zwijnaarde, Belgium) and normalized relative quantities were calculated by normalizing the data to the expression of previously validated endogenous control genes as described by Vandesompele et al. (2002). As housekeeping genes, *TATA-Box Binding Protein (TBP) and hypoxanthine phosphoribosyl transferase 1 (HPRT1)* were used. The normalized quantities were then determined for the candidate genes scaled against the expression values determined for the controls to generate fold changes in expression.

### Immunofluorescence

Immunofluorescence detection of inflammation- and fibrosis-associated markers was performed on cryosections of mouse sclera. After a 5 min rinse in PBS, slides were incubated for 1 h at room temperature (RT) in PBS containing 1% bovine serum albumin (Sigma-Aldrich, Vienna, Austria). Slides were subsequently incubated for double or triple immunohistochemistry (overnight at 4 °C) with antibodies directed against CD68 (NB100-683, Novus Biological, Colorado, USA, 1:50), COX2 (12282, Cell Signalling, Massachusetts, USA,1:200), IL6 (ab6672, Abcam, Cambridge, UK, 1:100), connective tissue growth factor (CTGF) (ab6992, Abcam, Cambridge, UK, 1:200), Matrix Metalloproteinase 2 (MMP2) (66366-1-Ig, Proteintech, Manchester, UK, 1:200), Matrix Metalloproteinase 3 (MMP3) (66338-1-Ig, Proteintech, Manchester, UK, 1:200), Matrix Metalloproteinase 13 (MMP13) (18165-1-AP, Proteintech, Manchester, UK, 1:200), Cleaved Caspase 3 (Asp175, #9661, Cell Signalling, Massachusetts, USA, 1:100). For all antibodies used, the manufacturers provided proof of validation in the antibody data sheets.

After a rinse in PBS (three times 5 min) binding sites of primary antibodies were visualized by corresponding Alexa 568- or Alexa 647-tagged antisera (1:500; Invitrogen, Karlsruhe, Germany) in PBS, containing 1% BSA (1 h at RT) followed by another rinse in PBS (three times 5 min). The GFP signal of the transgenic animals was enhanced using a goat anti-GFP antibody (GFP, #600-101-215S, Rockland, Limerick, USA; 1:500). The slides were counterstained for nuclei using DAPI. For that, slides were incubated for 10 min (1:4000, stock 1 mg/ml, VWR, Vienna, Austria) followed by a brief wash in PBS (three times 5 min). All slides were embedded in Fluoromount™ Aqueous Mounting Medium (Sigma-Aldrich, Vienna, Austria). Negative controls for background correction prior to semi-quantitative evaluation were performed by omission of the primary antibodies during incubation and resulted in absence of immunoreactivity. For semi-quantitative analysis, six sections of each animal were analysed, the mean values were used for statistical analysis (*n* = 3).

### Confocal imaging

Confocal images were acquired using a confocal laser scanning microscope (LSM710, Zeiss) equipped with 405, 488, 555, and 639 nm diode lasers, a 10 × EC Plan-Neofluar (10 × /0.3) or a 20 × Plan-Apochromat (20×/0.8) objective (Zeiss, Munich, Germany). Image acquisition was performed with the ZEN 2010 software (Zeiss), with image dimensions of 1024 × 1024 pixels and image depth of 16 bit. During image acquisition, two times averaging was applied and laser power and gain were adjusted to avoid saturation of single pixels. All images were acquired using identical microscope settings based on the control labelling of the secondary antibodies.

### Statistical analysis

Statistical analyses were performed using GraphPad Prism v.5.04 (La Jolla, CA, USA). Numerical data are presented as means ± standard deviation. For analysis of qPCR data, Mann–Whitney tests were performed, for semi-quantitative analysis of immunofluorescence stains Kruskal–Wallis tests for multiple comparisons were carried out. Statistical significance was set at α = 0.05.

## Results

**Fig. 1:**
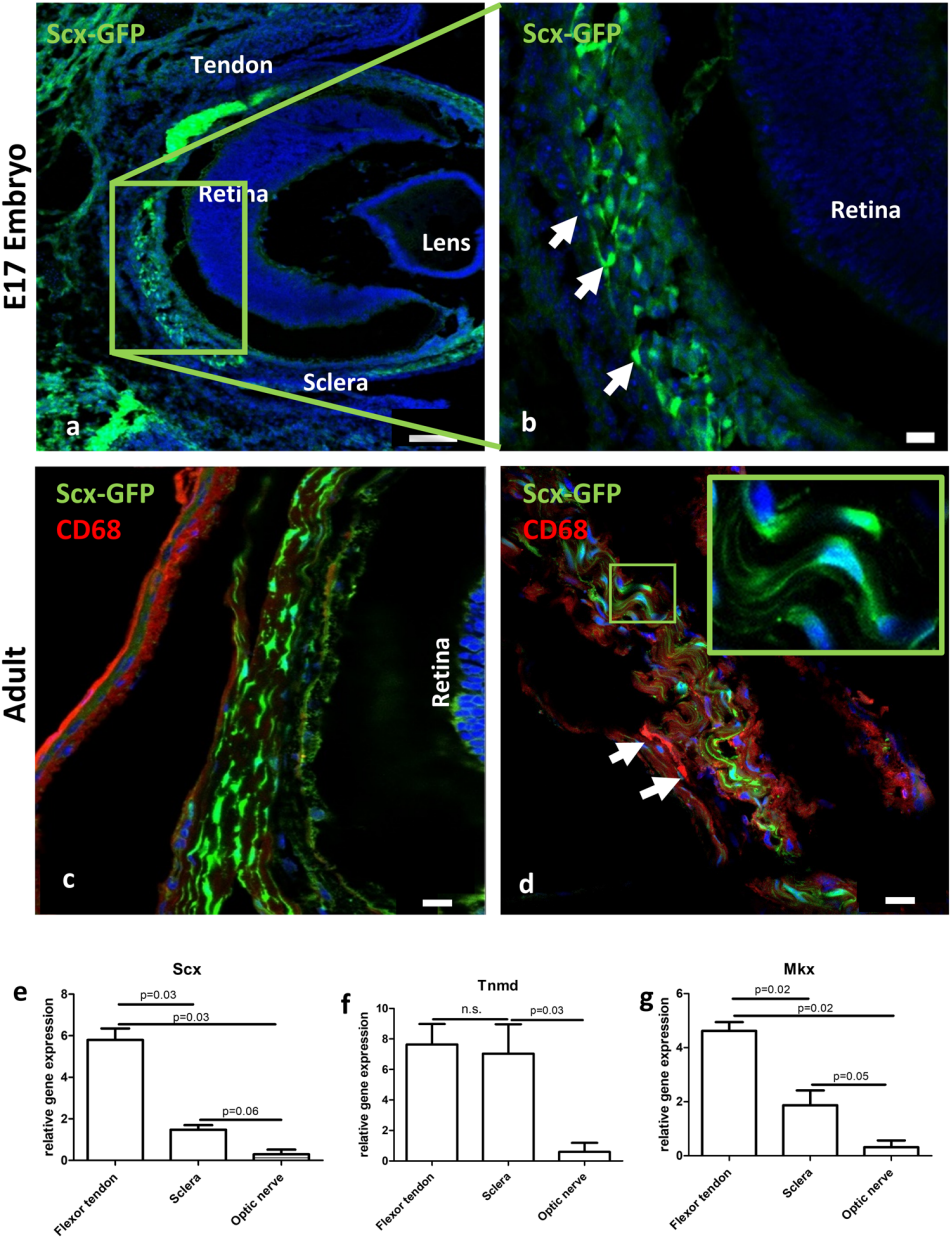
Scleral cells express tendon-associated markers. **a**, **b** Fluorescence microscopy revealed numerous scleraxis-GFP (Scx-GFP, *green*) positive cells in the sclera of embryonic mice at E17. **b** represents a magnification of the boxed area in a, highlighting the Scx-GFP positive cells (*arrows*). *Blue* = DAPI. **c**, **d** A similar situation was found in adult mice: spindle shaped Scx-GFP positive cells (*green*) were present throughout the entire sclera. Immunohistochemistry with CD68 revealed absence of co-localization with the Scx-GFP signal (**c**), while CD68-immunoreactivity was detected in few cells within the sclera (**d**, *arrows*). *Blue* = DAPI Scale bars: **a** = 100 µm, **b**–**d** = 20 µm. qRT-PCR analysis shows the expression of mRNA of the tendon-associated markers *scleraxis* (**e**), *tenomodulin* (Tnmd, **f**) and *mohawk *(Mkx, **g**), in both tendon and sclera. To a significantly lesser extent, expression is also detectable in the optic nerve.

Analysis of Scx-GFP reporter mouse eyes revealed a scleraxis immunopositive cell population in the sclera in both E17 embryos and adult animals (Fig. 1). The cells appeared spindle shaped with scarce cytoplasm, and cell diameters in the longitudinal axis of about 45 µm, and transverse axis of 15 µm, and with ovoid nucleus located in the center of the cell (Fig. 1d, inset). These GFP-positive cells located in all areas of the sclera with no obvious accumulation in a specific area. Double labelling with the macrophage marker CD68 revealed the presence of CD68^+^ cells in the sclera; however, these cells displayed no overlap with the Scx-GFP positive cell population (Fig. 1d). In addition, qRT-PCR analysis revealed expression of *scleraxis* (Fig. 1e), *tenomodulin* (Fig. 1f) and *mohawk*-mRNA (Fig. 1g) in tendon tissue as well as in the sclera. Also, in the optic nerve these markers are detectable, however to a significantly lesser extent.

**Fig. 2:**
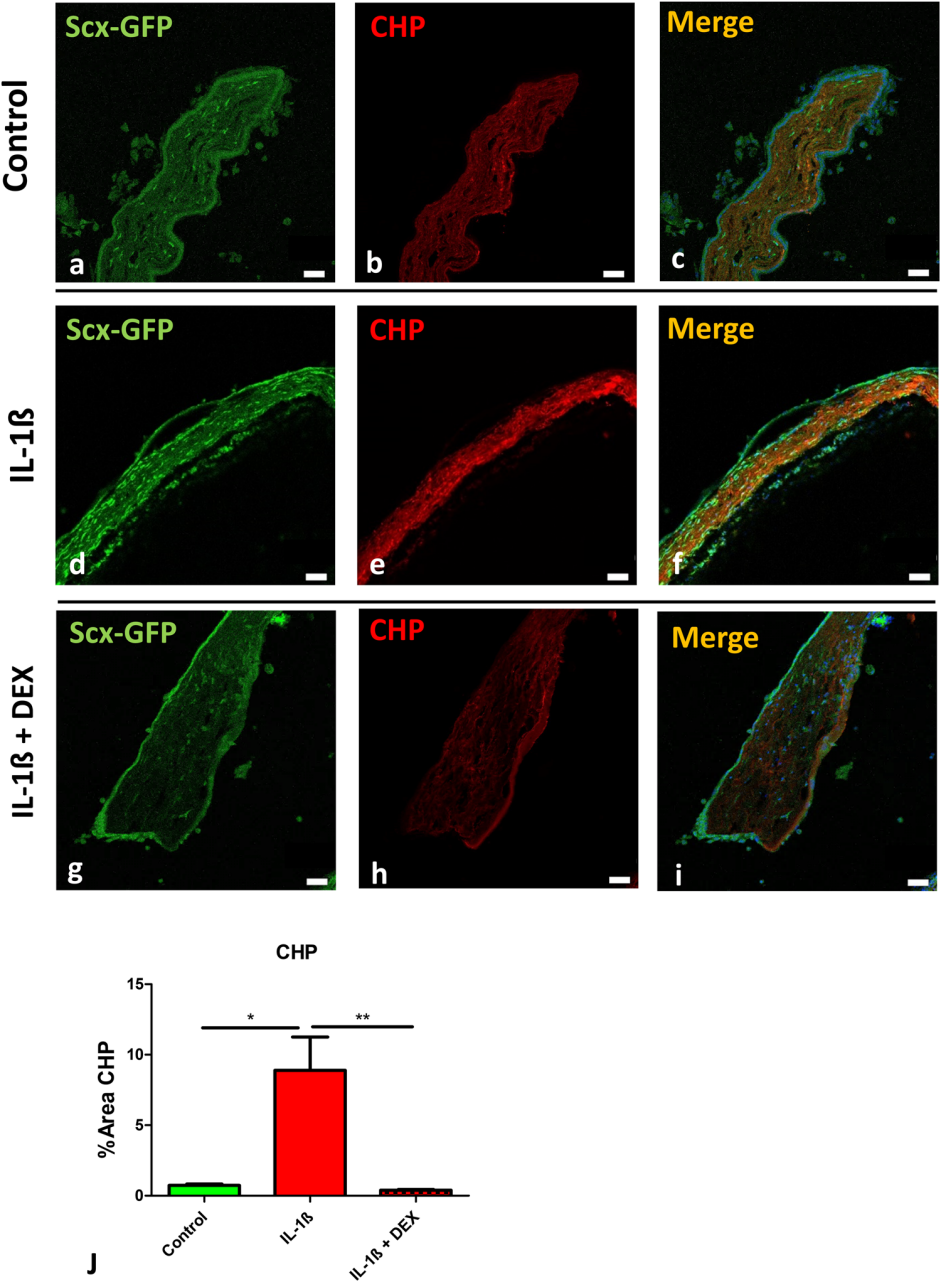
Interleukin treated scleral tissue undergoes collagen degradation. **a**–**i** Immunohistochemistry of Scx-GFP (*green*) and collagen hybridizing peptide (CHP, *red*, indicating collagen degradation). Compared to controls (**a**–**c**), a clear increase in signal intensity was evident in tissue incubated with IL1-ß (**d**–**f**) for both Scx-GFP and CHP that was abolished when treated with IL1-ß and dexamethasone (**g**–**h**). **j** Quantitative analysis of the signal increase of CHP **j** revealed statistical significance for IL-ß compared to IL1-ß and dexamethasone and control. Scale bars: 50 µm

In the organotypic tissue culture protocol, the addition of IL1-ß elicited a significant damage to scleral collagen, as seen by analysis of CHP binding. Quantification of the CHP positive area revealed an increase from 0.38% ± 0.25 in the control group compared to 8.89% ± 5.70 in the inflammatory-primed group (Fig. 2 a-f, j). To inhibit this effect, co-incubation experiments with dexamethasone were performed. As expected, CHP-positive areas were reduced to control levels (0.38% ± 0.14; Fig. 2g-i, j).

**Fig. 3:**
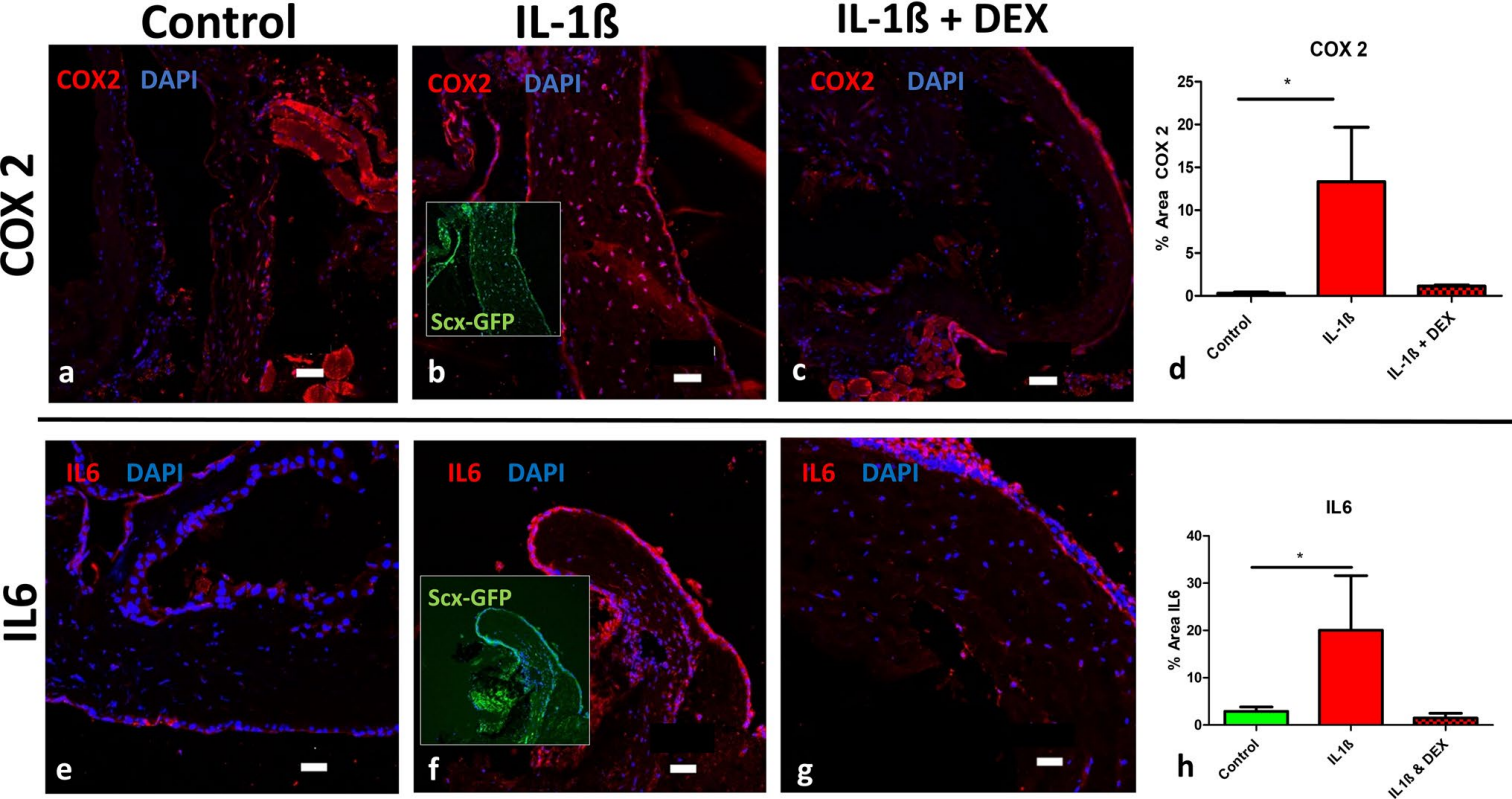
Interleukin treatment enhances the expression of COX2 and IL6 in scleral tissue. The inflammation-associated marker proteins COX2 (**a**–**d**) and IL6 (**e**–**h**) were increased when Scx-GFP mouse scleral tissue was incubated with IL1-ß compared to controls (**a**, **e**), and the effect is abrogated by addition of dexamethasone (**c**, **g**). Signal analysis revealed statistical significance for both COX2 (**d**) and IL6 (**h**) when incubated with IL-1ß compared to control. Scale bars: 50 µm

To verify the inflammatory response of Scx-GFP expressing scleral cells upon stimulation with IL1-ß, the expression of COX2 and IL6 was investigated. These experiments revealed an about 30-fold increase in expression in the experimental group (13.33% ± 11.01 vs. 0.31% ± 0.24 in the control samples), and again this effect was inhibited by the addition of dexamethasone (1.12% ± 0.24) (Fig. 3a–d) and IL6 (Fig. 3e–g) upon IL1-ß stimulation.

**Fig. 4:**
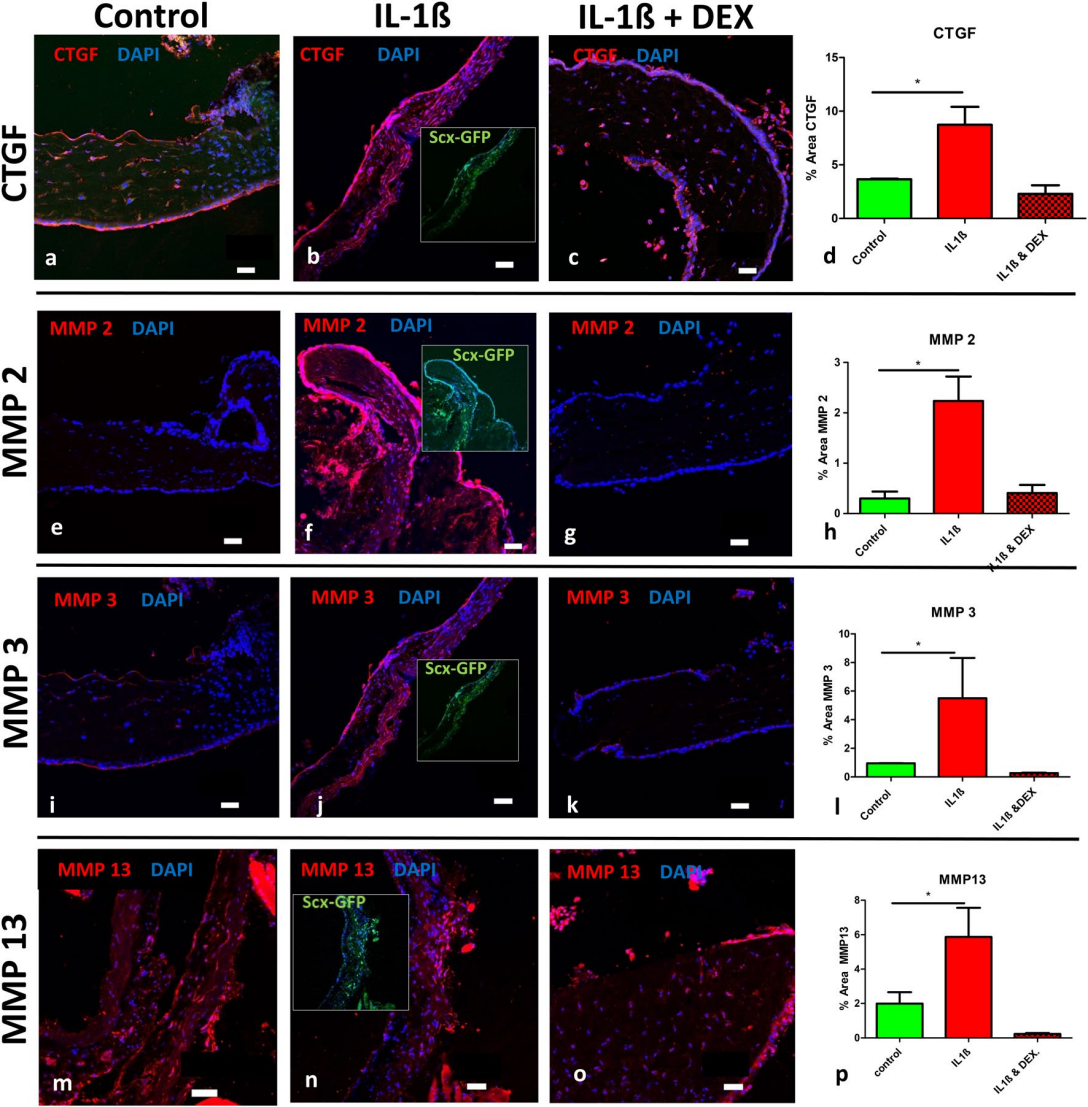
Interleukin treatment enhances the expression of fibrosis-associated proteins in scleral tissue. The fibrosis-associated marker protein Connective Tissue Growth Factor (CTGF, **a–d**), and matrix metalloproteinases MMP2 (**e**–**h**), MMP3 (**i**–**l**), and MMP13 (**m**–**p**) were increased in Scx-GFP mouse scleral tissue when stimulated with IL1-ß compared to controls, and the effects were abrogated by addition of dexamethasone (**c**, **g**, **k**, **o**). Signal analysis revealed statistical significance for all markers when incubated with IL-1ß compared to controls. Insets in the IL1-ß group images show the Scx-GFP channel and reveal co-expression of the respective proteins with Scx-GFP (insets in IL1-ß group). Scale bars: 50 µm

In a further set of experiments, the reaction of the fibrosis- associated marker connective tissue growth factor (CTGF) was investigated upon IL1-ß stimulation. Here, an increase in regions positive for expression due to IL1-ß treatment from 3.65% ± 0.10 to 8.73% ± 2.89 was observed, while this increase was reduced with addition of dexamethasone to 2.29% ± 1.39% (Fig. 4a–d). A similar response pattern was observed when investigating the change in expression of various matrix metalloproteases: MMP2 (control 0.29% ± 0.23, IL1-ß 2.24% ± 0.84, IL1-ß + Dexamethasone 0.41 ± 0.28, Fig. 4e–h), MMP3 (control 0.96% ± 0.01, IL1-ß 5.51% ± 4.87, IL1-ß + Dexamethasone 0.27% ± 0.04, Fig. 4i–l) and with MMP13 (control 1.99% ± 1.33, IL1-ß 5.87% ± 3.38, IL1-ß + Dexamethasone 0.23% ± 0.11, Fig. 4m–p).

**Fig. 5:**
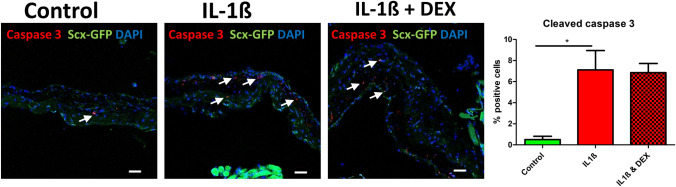
Interleukin treatment induces apoptosis in scleral cells. The apoptosis-associated marker protein Cleaved caspase 3 was increased in Scx-GFP mouse scleral tissue when stimulated with IL1-ß compared to controls, dexamethasone did not abrogate this effect (**a**–**d**). Scale bar: 50 µm

Treatment of scleral samples with 10 ng/ml IL1-ß induced apoptosis to a significant extent (control 0.50% ± 0.71, IL1-ß 7.11% ± 4.48, IL1-ß + Dexamethasone 6.86 ± 1.71), with no rescue effect caused by addition of dexamethasone (Fig. 5).

## Discussion

We here describe a population of cells in the sclera expressing tendon-related markers responding to inflammatory stimulation.

By size and shape, the Scleraxis-positive scleral cells rather resemble fibroblasts. Since they do not express CD68, they do not seem to belong to macrophage-like cells. The observed presence of CD68 + scleral macrophages (Fig. 1d) is in line with previous reports showing macrophages in human and mouse sclera (Xu et al. 2007; Schlereth et al. 2016). The observed expression of tenomodulin and mohawk-mRNA further substantiates the presence of cells expressing tendon-related markers in scleral tissue.

Regarding the expression of tendon related markers in the sclera, our observation of Scleraxis- positive cells is in line with reports on tenomodulin expression in the sclera, a presumably antiangiogenic factor also expressed by tendon cells (Oshima et al. 2003).

There are relatively few studies that have systematically studied the histopathology of scleritis, as a scleral biopsy is not commonly performed for diagnostic purposes due to the risks of worsening the inflammatory process or structurally weakening an already compromised organ. Most information on structural and histological alterations in eyes affected by scleritis stem from eyes removed surgically because they were blind and painful, or from eyes obtained at autopsy(Hankins and Margo 2019). Therefore, particularly early events in this disease and immunohistochemical aspects are not well studied.

In tendon, we could show that cells respond to stimulation with IL1-ß by upregulation of inflammatory and fibrotic marker genes in vitro (Lehner et al. 2019). Similarly, the Scx-GFP positive scleral cells upregulate COX2 and IL6.

The observed upregulation of CTFG indicates a fibrosis-like response to stimulation with IL1-ß, as it has been described in fibrotic tendon (Morita et al. 2016). In the sclera, CTGF was shown to be regulated in recovering experimental myopia, with a downregulation during a hyperopic refractive error causing GO signals, whereas it was found to be upregulated in the remodelling process during recovery (Guo et al. 2014).

Regarding the involvement of matrix metalloproteinases in scleritis, relatively little is known so far. Both MMP2 and MMP3 were shown to be expressed by resident scleral fibroblasts as well as inflammatory cells such as macrophages and T lymphocytes in necrotizing scleritis (Di Girolamo et al. 1997). MMP2 was found to be expressed in samples of human melanoma-associated spongiform scleropathy, whereas MMP13 could not be detected in this study (Alyahya et al. 2008). In tendon, MMPs are well described to be involved in matrix remodelling and degeneration in tendinopathy, causing collagen fibre disruption and tissue weakening (Riley 2008).

The observation of apoptosis induction in scleral cells by IL1-ß is in line with our previous findings of tendon cells undergoing apoptosis following stimulation by 10 ng IL1-ß/ml (Gehwolf et al. 2019). The fact that dexamethasone does not significantly rescue scleral cells from IL1-ß induced apoptosis in our hands may be due to ambivalent influence of dexamethasone on apoptosis. In proliferative chondrocytes, dexamethasone has been shown to induce apoptosis itself via activation of caspases (Chrysis et al. 2005). Interestingly, several other cell types, such as neutrophils or keratinocytes have an anti-apoptotic response to glucocorticoids that is cytoprotective (Gruver-Yates and Cidlowski 2013).

A limitation of this study is the indirect proof of scleraxis expression in the sclera by a transgenic animal model. In our hands, no antibody was sufficiently specific to credibly label scleraxis by immunohistochemistry. Therefore, also the intensity of the scleraxis-GFP signal should not be over-interpreted.

However, with this work, we introduce a novel organotypic in vitro model of scleritis, using a scleraxis-GFP reporter mouse model, and provide a first characterization of these tendon marker expressing scleral cells in response to inflammatory stimulation.

Summarizing, with this study, we show that Scleraxis-positive scleral fibroblasts respond to inflammatory stimulation in a similar fashion as it has been demonstrated for tendon. Moreover, we establish a novel ex vivo model of scleritis, leading to hallmarks of this disease like damaged collagen matrix, upregulation of inflammatory factors as well as to the expression of matrix degrading enzymes.

## Supplementary Information

Below is the link to the electronic supplementary material.

Supplementary Fig 1 Schematic figure for the organotypic sclera inflammation model experiment as described in the material and methods section. Freshly isolated scleral tissue of adult female Scleraxis-GFP (Scx-GFP) mice (*n*= 3 per group) were removed and 3 equally sized pieces were immediately transferred for 3 days at 37°C into control medium (green arrow), medium containing IL1-ß (red arrow), and medium containing IL1-ß and dexamethasone (checkers arrow), respectively. The tissues were processed for cryosectioning and analyzed by immunofluorescence for inflammation- and fibrosis-associated proteins (Supplementary file1 TIF 6848 kb)

Supplementary Fig 2 Merged images of Scx-GFP visualization and immunostainings of inflammation- and fibrosis associated proteins show expression of these markers in Scx-GFP+ cells. Scale bar: 50µm (Supplementary file 2 TIF 14423 kb)

## Data Availability

All data and materials are available on reasonable request.
